# Monitoring and Modeling of Emissions from Concentrated Animal Feeding Operations: Overview of Methods

**DOI:** 10.1289/ehp.8838

**Published:** 2006-11-14

**Authors:** Bryan Bunton, Patrick O’Shaughnessy, Sean Fitzsimmons, John Gering, Stephen Hoff, Merete Lyngbye, Peter S. Thorne, Jeffrey Wasson, Mark Werner

**Affiliations:** 1 Iowa Department of Natural Resources, Des Moines, Iowa, USA; 2 College of Public Health, The University of Iowa, Iowa City, Iowa, USA; 3 Iowa State University, Ames, Iowa, USA; 4 The National Committee for Pig Production, Copenhagen, Denmark; 5 University Hygienic Laboratory, Iowa City, Iowa, USA; 6 Wisconsin Department of Health and Family Services, Madison, Wisconsin, USA

**Keywords:** ammonia, animal feeding operation, dispersion model, hydrogen sulfide, monitor, odor, particulate matter, poultry, swine

## Abstract

Accurate monitors are required to determine ambient concentration levels of contaminants emanating from concentrated animal feeding operations (CAFOs), and accurate models are required to indicate the spatial variability of concentrations over regions affected by CAFOs. A thorough understanding of the spatial and temporal variability of concentration levels could then be associated with locations of healthy individuals or subjects with respiratory ailments to statistically link the presence of CAFOs to the prevalence of ill health effects in local populations. This workgroup report, which was part of the Conference on Environmental Health Impacts of Concentrated Animal Feeding Operations: Anticipating Hazards—Searching for Solutions, describes instrumentation currently available for assessing contaminant concentration levels in the vicinity of CAFOs and reviews plume dispersion models that may be used to estimate concentration levels spatially. Recommendations for further research with respect to ambient air monitoring include accurately determining long-term average concentrations for a region under the influence of CAFO emissions using a combination of instruments based on accuracy, cost, and sampling duration. In addition, development of instruments capable of accurately quantifying adsorbed gases and volatile organic compounds is needed. Further research with respect to plume dispersion models includes identifying and validating the most applicable model for use in predicting downwind concentrations from CAFOs. Additional data are needed to obtain reliable emission rates from CAFOs.

## Background and Recent Developments

Airborne contaminant emissions emanating from concentrated animal feeding operations (CAFOs) include toxic gases and particulates. A combination of gases or particulates of sufficient concentration and chemical composition may also be perceived as an irritant odor downwind of a CAFO. There is a need to accurately assess the concentrations of these contaminants in order to determine the effect they may have on the health of residents living in proximity to CAFOs.

A variety of analytical methods are available for measuring toxic gases, particulates, and odor and vary significantly in terms of cost, precision, accuracy, portability, maintenance requirements, and the ability to conduct continuous measurements. When using these methods, both the spatial and temporal variability of the pollutant concentrations must be considered. For example, the temporal variability for a certain location can be measured with a direct-reading instrument, but this will not indicate the level of a contaminant over a broad area. Instruments of this type tend to be accurate but expensive. Similarly, time-integrating samplers, which typically are inexpensive but lack accuracy, can be deployed over a large area to assess the spatial variability of a pollutant. In a recent study conducted in the United States, air pollution problems caused by emissions from animal feeding operations into ambient air were characterized as “local” scale if neighbors living near the animal feeding operation were affected, or “regional” or “national” scale if the pollution emitted affects the quality of life in a multistate or national area (National Research Council Ad Hoc Committee on Air Emissions from Animal Feeding Operations, Committee on Animal Nutrition 2003). Ammonia was classified as a major pollutant on the regional scale, whereas hydrogen sulfide, particulate matter, and odor were classified as major pollutants at the local scale.

Another method for determining the spatial variability of an airborne contaminant is to utilize a plume dispersion model. A model of this type can develop concentration isopleths over an area from weather data averaged over a variety of time periods such as a day, month or year. The corollary approach is to use a spatial interpolation method such as kriging (Carletti et al. 2000; Zirschky 1985) to formulate concentration isopleths from measurements derived from time-integrated samplers. However, this review focuses on the instruments available for measuring gases, particulates, and odor, and on plume dispersion models applicable to the determination of contaminants in the vicinity of CAFOs.

### Ammonia monitoring

Relatively accurate but expensive instruments (> US$10,000) are available for measuring ammonia and hydrogen sulfide at the limit of detection (parts per billion level) needed to determine the low concentrations of these gases expected at the local scale. There are several types of detection devices available for each gas, including chemiluminescence analyzers for oxides of nitrogen (NO_x_) and pulsed fluorescence analyzers for sulfur dioxide (SO_2_) that are commonly used for ambient air quality monitoring and contain thermal oxidizers for the quantification of ammonia (NH_3_) and hydrogen sulfide (H_2_S), respectively (Thermo Electron Inc., Waltham, MA, USA). The U.S. Environmental Protection Agency (U.S. EPA) guidance for continuous reference methods dictates that these monitors must be operated between 20 and 30°C, necessitating a temperature-controlled enclosure at the monitoring site. McCulloch and Shendrikar (2000) concluded that an ammonia analyzer of this type reliably measured hourly ambient ammonia concentrations with a high degree of accuracy.

Ammonia monitors based on a photo-acoustic infrared absorption technique are also in current use (Innova Air Tech Instruments, Ballerup, Denmark; Pranalytica Corporation, Santa Monica, CA, USA). The ammonia molecule has infrared absorption bands, and pulses of infrared radiation can be converted to pressure waves in a measurement cell containing ammonia (Pushkarsky et al. 2002). Correcting for infrared absorption by water vapor and carbon dioxide allows for quantification of ammonia concentrations. A design that has been widely used in the Netherlands for ambient ammonia monitoring involves the use of a semipermeable membrane (Mechatronics Instruments, Hoorn, the Netherlands). The membrane selectively passes gaseous ammonia, which is then absorbed into a liquid reagent inside the sampler. A conductivity detector records the changing conductivity of the reagent solution, which is proportional to the ammonia concentration (Erisman et al. 2001). This measurement method compared favorably with others in a European study (Mennen et al. 1996). Another design incorporating a semipermeable membrane and a special liquid reagent has been proposed by Li et al. (1999). After the reagent reacts with ammonia, it forms a compound (1-sulfonatoisoindole), which when illuminated with ultraviolet light, fluoresces at a known wavelength. A photodiode records the amount of fluorescent light, which is proportional to the ammonia concentration. A fluorimetric enzyme method with a limit of detection of 110 μg/L was used to measure ambient ammonia levels in the vicinity of a swine facility (Subramanian et al. 1996).

Open-path monitoring methods for ammonia are commercially available and have been used extensively by the state of Missouri and the U.S. EPA for investigative surveys and emission factor development near CAFOs (Childers et al. 2001; Harris et al. 2001; Hashmonay et al. 1999a, 1999b). Open-path monitoring methods measure the absorption of light as the light beam traverses the path between the light source and a reflector. The absorption spectra obtained from these instruments is used to uniquely identify ammonia among other light-absorbing gases, and the amount of absorption measured may be used to determine the average ammonia concentration along the path. An open-path monitor produces path-average concentrations, and this average may be higher or lower than the concentrations measured at particular points along the path.

At the regional scale, a nationwide monitoring network designed to measure the deposition of ammonia and other ions in rainfall has been constructed as part of the National Atmospheric Deposition Program. Atmospheric deposition of ammonia or ammonium gives rise to the eutrophication of ecosystems (Bouwman and Van Vuuren 1999; Burkholder et al. 2006; Sheppard 2002). Ammonium nitrate and ammonium sulfate are also significant contributors to regional fine particulate pollution problems present in California and in parts of the eastern and southeastern United States (U.S. EPA 2003a) and contribute to visibility reduction at national parks in the United States (National Research Council Committee on Haze in National Parks and Wilderness Areas 1993). The U.S. EPA also funds the operation of a nationwide network of fine particulate speciation samplers known as the Speciation Trends Network. Chemical analyses of filters from these samplers are used to establish levels of ammonium sulfate and ammonium nitrate and other constituents of fine particulate. The National Park Service operates a similar nationwide network of speciated fine particulate samplers as part of the Interagency Monitoring of Protected Visual Environments (IMPROVE) Program.

### Hydrogen sulfide monitoring

A variety of analytical methods ranging in cost from US$5,000 to US$20,000 are available for measuring hydrogen sulfide. Parbst et al. (2000) has compared the variability obtained from different portable and nonportable hydrogen sulfide monitoring equipment. Some agricultural states, including Nebraska, Minnesota, and Missouri, have established their own air quality standards for hydrogen sulfide or total reduced sulfur. Monitoring networks established by these states use pulsed fluorescence analyzers to determine subhourly concentrations for comparison with state standards (State of Colorado 1999; State of Minnesota 2003; State of Missouri 2004; State of Nebraska 2002).

One type of portable hydrogen sulfide monitor determines hydrogen sulfide concentrations from changes in conductivity across a gold film (Arizona Instruments, Tempe, AZ, USA). The conductivity of the film varies as a function of the amount of hydrogen sulfide that has been deposited on its surface. A second portable monitoring design relies on the color change of a chemically treated tape as it is exposed to hydrogen sulfide. One type of tape used to measure hydrogen sulfide is coated with lead acetate. Upon exposure to hydrogen sulfide, a color change occurs as a result of the formation of lead sulfide. A recent study (Campagna et al. 2004) of ambient and indoor hydrogen sulfide levels in a community in Nebraska used a proprietary tape-based monitor (Zellweger Analytics, League City, TX, USA). A monitor of this design has been approved for hydrogen sulfide monitoring in Minnesota. Toda et al. (2001) have performed interference and sensitivity tests involving gold-film and tape-based hydrogen sulfide monitoring methods.

### Odor measurements

The quantitation of odor is more challenging because it represents a varying complex mixture of free and particle-bound compounds. An ideal approach to odor measurement would begin by characterizing the chemical constituents associated with a particular offensive odor. Odors could then be quantified objectively based on the identification and quantification of the speciated constituents. However, the correlation between human response and specific compounds identified by instrumental methods such as gas chromatography remains quite poor (Powers et al. 2000). One possible explanation is that the human nose may be sensitive to concentrations that lie below instrumental detection limits. Also, the simultaneous instrumental determination of more than 200 compounds that have been identified in livestock odors is difficult because different groups of compounds require different types of columns for efficient separation as well as different operating parameters and detectors. Currently, existing limitations of instrumental methods make the human observer a necessary part of the odor measurement methods. Livestock odor measurement techniques currently rely on trained human raters for odor quantification. In Colorado and Missouri, measurements are taken using a scentometer, a simple portable device used to dilute odorous air with odor-free air (Barnebey Sutcliffe Corp., Columbus, OH, USA, and St. Croix Sensory, Inc., Lake Elmo, MN, USA) (State of Colorado 1999; State of Missouri 2003). A more accepted method for odor assessment is olfactometry, which has been established as American Standards of Testing and Measurements (ASTM) methods E1432-91 and E679-91 (ASTM 1997a, 1997b respectively). Odorous air samples are taken in the field using Tedlar bags, and returned to the laboratory for evaluation by a trained panel of odor observers using a device known as an olfactometer (St. Croix Sensory Inc.), which allows the panel members to sniff increasing dilutions of the odorous air randomly delivered to one port or another with odor-free air until no odor can be detected. Comparability of odor measurements taken with a scentometer with data taken with an olfactometer is problematic (Bottcher 2001). While olfactometers filter out particulate matter, filters are not used in commercially available scentometers. Scentometers, on the other hand, have limited resolution. It has been reported that an important fraction of odorous material may adhere to the surface of particulate matter (Powers et al. 2000).

### Particulate matter monitoring

Particles may act as carriers for microorganisms and endotoxin, adsorbed gases and vapors such as ammonia, and a variety of compounds that contribute to odor. Instrumentation for the measurement of ambient particulate concentrations are well developed and have been in use for decades as part of national ambient air quality sampling networks. Typical gravimetric instruments used for this purpose draw air through a large filter at a high flow rate (1–40 cfm or 30–1,200 L/min). Air inlets are often applied to segregate the smaller, potentially more harmful, particles. Information on the use of these instruments can be found through the Technology Transfer Network of the U.S. EPA (U.S. EPA 2003a). Direct-reading particulate monitors are also available (Watson et al. 1998). Although numerous studies have been conducted to indicate particulate concentrations within CAFOs, very few have involved the detection of dust levels downwind of these facilities, perhaps because of the many sources for dust in rural areas, such as unpaved roads, that would confound results.

### Dispersion models

Dispersion models were first approved for regulatory application in 1977 when they were incorporated into the Clean Air Act (Jacobson et al. 1999). Since that time, the state of science of dispersion modeling has improved greatly. Dispersion models are a set of mathematical equations that attempt to simulate (model) the transport, diffusion, chemical transformation, physical interactions, and removal of pollutants in the atmosphere. Typically, model solutions are expressed as concentrations for some time period at “receptor” locations. Currently, the U.S. EPA has approved a number of models for regulatory application, and lists them in Appendix A of the *Guideline on Air Quality Models* [published as Appendix W of 40 CFR Part 51 (U.S. EPA 1998)]. These are divided into three categories: preferred or recommended refined dispersion models, screening models that can precede the use of a refined modeling analysis, and refined air quality models for use on a case-by-case basis for individual regulatory applications. In addition to models developed by the U.S. EPA, proprietary or research models have been developed to examine pollutant dispersion for specific needs.

Various inputs into these models include the source type such as point, line, area, pit, or volume, and source data such as location, gas temperature and velocity, and pollutant release rate (mass/time). In addition, hourly meteorologic data are added to these models and should contain wind speed and direction, ambient air temperature, stability class, mixing height, and precipitation, and pressure. Some models also contain options for inclusion of chemical transformation of gases. Regional models may contain inputs for geophysical data, such as terrain elevations and land use, surface and upper air meteorology, precipitation data, cloud observations, and visibility and deposition flux calculations.

Models can be applied for analysis of dispersion on both a local and regional scale. Local scale dispersion modeling usually predicts concentrations in an area < 50 km, and determines ambient impacts from one or more facilities. For assessing short-range transport of pollutants, the U.S. EPA recommends the Industrial Source Complex Short-Term Model, version 3 (ISC-ST3) (U.S. EPA 1995). The ISC-ST3 model is a steady-state, Gaussian plume model suitable for a wide range of industrial applications and special cases. Models of this type operate under the assumption that the contaminant disperses from a source with a concentration profile defined by a normal or Gaussian curve. It should be noted that the U.S. EPA recommended a new Gaussian plume model, the American Meteorological Society/Environmental Protection Agency Regulatory Model (AERMOD), for regulatory applications in November of 2005. Both ISC-ST3 and AERMOD may be used for regulatory purposes during a one year transitional period ending in November 2006, at which time AERMOD will become the U.S. EPA recommended model (U.S. EPA 2005).

Regional modeling is used to calculate pollutant concentrations over a much greater area (> 50 km). For assessing the long range transport of pollutants, the U.S. EPA recommends the use of CALPUFF, a non-steady-state Lagrangian puff dispersion model that depends on high-definition meteorologic data. Models of this type can account for an intermittent release rather than assuming a steady, continuous stream by simulating pollutant releases as a continuous series of puffs. More detailed applications of regional pollutant modeling are conducted with photo-chemical models that incorporate detailed atmospheric chemistry processes on large scales. Examples of these models include the Comprehensive Air Quality Model with extensions (CAMx) model maintained by the Environ Corporation (Newark, NJ, USA), the Community Modeling Air Quality (CMAQ) model developed by the U.S. EPA, and the Fine Resolution Atmospheric Multi-Pollutant Exchange (FRAME) regional model, developed specifically to describe atmospheric transport and deposition of ammonia (Singles et al. 1998). While these models include sophisticated chemical and physical processes, application for local-scale studies likely will be problematic because of the grid cell-level dilution.

### Modeling pollutants emitted from CAFOs

Attempts have been made to use air dispersion models to estimate concentrations of both odor and contaminants downwind of CAFOs. These studies are complicated by three important factors: there may be several sources of a contaminant; the emission rate from each source is difficult to precisely determine; and the regulatory models do not typically include provisions for the degradation and deposition of gases in transport downwind from the source.

The ISC-ST3 model has been recommended for estimating air quality impacts of feedlot operations (Earth Tech 2001). However, ISC-ST3 has known deficiencies during stable or near-calm conditions when CAFO odors are most offensive, and it cannot directly account for effects of vegetation on concentrations or small-scale effects of terrain on the wind fields. In addition, Gaussian plume models may not adequately predict concentrations of compounds that are heavier than air, such as hydrogen sulfide, a pollutant of concern near animal feeding operations. Modifications to the Gaussian plume model that better represent agricultural sources have been investigated (Gassman and Bouzaher 1995; Keddie 1980; Rege and Tock 1996). A detailed discussion of transport from ground-level agricultural sources can be found in Smith (1993) with an emphasis on the dispersion of particulates from low-level sources given by Fritz et al. (2002). The AERMOD model may have particular applicability to modeling emissions from animal agriculture by including the air boundary layer above surface releases, such as from manure storage basins (Jacobson et al. 1999). There have also been efforts to use computational fluid dynamics to model the dispersion of contaminants from agricultural buildings (Quinn et al. 2001). These need further development.

CALPUFF has recently been used to model ammonia and hydrogen sulfide in the vicinity of a CAFO (Minnesota Pollution Control Agency 2003). Some of the attributes of CALPUFF are especially pertinent to conditions associated with CAFOs: variable wind directions, calm-wind algorithm, buoyant area and line sources, nonuniform land patterns, and multifacility applications. The state of Minnesota has opted to use the ISC-ST3 model for single facilities and the CALPUFF model for multi-facility applications (Earth Tech 2001) and the U.S. EPA has adopted CALPUFF as the preferred model for assessing long-range transport of pollutants (U.S. EPA 2003b).

Other studies have focused on the dispersion of odor primarily to determine setback distances between CAFOs and local residences (Heber 1997; Jacobson et al. 2001; Zhu et al. 2000). Gaussian plume models that have been widely used for odor dispersion modeling include the Australian Plume (AUSPLUME) model (Environmental Protection Authority–Victoria 2000), ISC-ST3 (U.S. EPA 1995), and STINK (Smith and Watts 1994). Studies have shown varying degrees of agreement between model results and odor measurements (Carney and Dodd 1989; Gassman 1992; Guo et al. 2001; Li et al. 1994). Gassman (1992) reviewed literature on odor modeling using the Gaussian plume method and concluded that the method was best applied on a relative basis for comparing differences between different facilities. Koppolu et al. (2002) compared results obtained from AERMOD and STINK after modeling the dispersion of low molecular weight volatile odorous fatty acids. They found better agreement between model results and measured values when using AERMOD. The Gaussian Integrated Puff (INPUFF-2) model has also been used to predict odor dispersion (Zhu et al. 2000).

## Workshop Recommendations

### Priority research needs

- Monitoring networks: There is a need to accurately determine long-term average concentrations for a region under the influence of CAFO emissions using a combination of instruments based on accuracy, cost, and sampling duration. This may include an array of passive monitors in combination with occasional spot-checks using portable devices, co-located instruments and laboratory testing for quality control. Passive monitors for hydrogen sulfide are available with detection limits of 0.1 ppb (Radiello Inc., Italy; MAXXAM Analytics, Inc., Calgary, Alberta, CN). Information of this type could be used to correlate contaminant exposures to chronic health effects through the use of community surveys, questionnaires, medical examinations, or other validated methods with contaminant exposures. Similar correlations could be made using existing direct-reading instruments to gather short-term data coincidental with acute effects. Monitoring networks have been established to measure gases and odors in the vicinity of CAFOs, but few have incorporated particulate measurements, especially the components of particles that contribute to odor such as adsorbed gases and volatile organic compounds. Instruments are needed to accurately detect these compounds. Furthermore, spatiotemporal geostatistical techniques for accurately placing monitors and interpolating concentration measurements derived from monitoring networks are needed. Similarly, the integration of data from a variety of sources such as model predictions, satellite data, and monitored values should be explored.
- Background levels: Research is needed to determine background levels in rural areas as well as to obtain a better understanding of concentrations inside residences and schools relative to outdoor concentrations. This work should also include an assessment of how far gases and particulates travel from CAFOs to determine their area of influence.
- Causative agents: Epidemiologic studies are needed to determine the actual causative agents related to health outcomes to ensure that the most-important contaminants are being measured. A metric to relate odor with health symptoms is needed.
- Model selection: Research should be conducted to identify and validate the most applicable model for CAFO emissions. Specifically, models that account for the chemical transformation of pollutants, such as ammonia and hydrogen sulfide, are needed. Model accuracy should be evaluated and prediction error determined through comparison of predicted values with actual monitoring data. In addition, future research that involves dispersion modeling is needed to assess public health concerns by determining long-term concentrations within a region, providing exposure data for health outcomes research, assessing meteorologic conditions during the past that could induce events, and forecasting future events. There is further need for models that will enable evaluation of concentration/exposure scenarios after an event that triggered hospital visits (e.g., asthma attack) and nuisance complaints.
- Toxicant emissions: Tied to the development of accurate dispersion models is the need for improved understanding of the rate of contaminant emissions from CAFOs. Research is needed to obtain reliable emission rates that consider temporal variations and the influence of management practices in addition to current knowledge concerning emissions related to the CAFO type, the number of animals, and the manure storage and handling.

#### Translation of science to policy

Modeling is an important tool for use in regulatory applications regarding industrial sources, but its use should be expanded to CAFOs. For existing livestock facilities, modeling could be used to determine the results of best management practices through estimation of potential reductions or expected concentrations on downwind receptors. For proposed livestock facilities, modeling could be used by local zoning officials or state or federal regulatory agencies to assess potential impacts on surrounding rural populations or environmentally sensitive areas prior to construction. Additionally, the use of modeling could enable policy makers to establish setback distances prior to construction of a livestock facility that are based on predicted concentration profiles, and would therefore be protective of public health. Finally, modeling could be used to survey situations affecting a single community, eliminating some across the board regulations for more of a case-by-case approach.
